# Different lesion distribution in calves orally or intratracheally challenged with *Mycobacterium bovis*: implications for diagnosis

**DOI:** 10.1186/s13567-018-0566-2

**Published:** 2018-07-27

**Authors:** Miriam Serrano, Iker A. Sevilla, Miguel Fuertes, Mariví Geijo, Maria Ángeles Risalde, Jose Francisco Ruiz-Fons, Christian Gortazar, Ramón A. Juste, Lucas Domínguez, Natalia Elguezabal, Joseba M. Garrido

**Affiliations:** 1 Animal Health Department, NEIKER-Instituto Vasco de Investigación y Desarrollo Agrario, Derio, Bizkaia Spain; 2 SaBio (Health and Biotechnology), Instituto de Investigación en Recursos Cinegéticos IREC (CSIC-UCLM), Ciudad Real, Spain; 3 Dpto. de Anatomía y Anatomía Patológica Comparadas, Agrifood Campus of International Excellence (ceia3), Universidad de Córdoba, Córdoba, Spain; 4 VISAVET Health Surveillance Centre, Complutense University of Madrid, Madrid, Spain; 5 Present Address: SERIDA, Agri-food Research and Development Regional Service, Villaviciosa, Asturias, Spain

## Abstract

Animal tuberculosis (TB) remains a major problem in some countries despite the existence of control programmes focused mainly on cattle. In this species, aerogenous transmission is accepted as the most frequent infection route, affecting mainly the respiratory system. Under the hypothesis that the oral route could be playing a more relevant role in transmission, diagnosis and disease persistence than previously thought, this study was performed to assess the course of TB infection in cattle and its effects on diagnosis depending on the route of entry of *Mycobacterium bovis*. Two groups of five calves each were either endotracheally (EC) or orally (OC) challenged. Necropsies were carried out 12 weeks after challenge except for three OC calves slaughtered 8 weeks later. All animals reacted to the tuberculin skin test and the entire EC group was positive to the interferon-gamma release assay (IGRA) 2 weeks after challenge and thereafter. The first positive IGRA results for OC calves (3/5) were recorded 4 weeks after challenge. Group comparison revealed significant differences in lesion and positive culture location and scoring. TB-compatible gross lesions and positive cultures were more frequently found in the thorax (*p* < 0.001) and lung (*p* < 0.05) of EC animals, whereas OC animals presented lesions (*p* = 0.23) and positive cultures (*p* < 0.05) mainly located in the abdomen. These results indicate that the infection route seems to be a determining factor for both the distribution and the time needed for the development of visible lesions. Our study suggests that confirmation of TB infection in some skin reactor animals can be problematic if current post-mortem examination and diagnostics are not improved.

## Introduction

*Mycobacterium bovis* is the main etiological agent of animal tuberculosis (TB), a mycobacterial infectious disease with a worldwide distribution [1] that affects cattle [2], other domestic hosts [3], wildlife [4] and humans [5]. The huge economic losses caused by bovine TB added to the impact of its zoonotic nature led to implement control strategies for over a century in many countries [6, 7]. Although eradication of TB has been accomplished in some countries, the presence of *M*. *bovis* in herds continues to pose serious problems for animal and human health in many others [7–11]. This discrepancy has been observed despite the similarity of the eradication programs used in the different countries [12, 13]. There are several reasons for the persistence of the disease in cattle, but it is usually attributed to the existence of wild reservoirs [4]. Domestic reservoirs include goats [14], sheep [15] and pigs [16], depending on the characteristics of the local host community.

Different transmission pathways do exist for cattle. These include direct or indirect inhalation, oropharyngeal exposure and/or ingestion of *M. bovis* and, more unlikely because of the active eradication programs, transplacental or mammary transmission [17]. Lesion distribution and progression seem to be shaped by the route of introduction of the bacterium [18, 19]. There is a general acceptance that the aerogenous transmission is the most frequent one in cattle and lesions are usually found in the respiratory system and associated lymph nodes (LN) [17, 18]. This also seems to be the case for natural intra-species transmission in the badger, the principal wild animal reservoir in Ireland and UK [20]. Lesions can also reach these LN and other tissues or LN of the head region [17, 18, 21] after oral exposure to *M*. *bovis*. However, ingestion of bacilli is usually associated with affected LN and tissues of the digestive system with or without visible lesions [17, 18]. Oral exposure to *M*. *bovis* could represent a more relevant route of infection than previously thought. In the wildlife-livestock interface inter-species transmission is of an indirect nature, for instance through shared water or food [22, 23]. In these cases, infection will most likely enter the host by the oral route. Widespread contamination of environmental samples in the Iberian Peninsula suggests that indirect transmission contributes to the maintenance of tuberculosis in multi-host–pathogen systems [24, 25].

Cell mediated immunity (CMI)-based diagnostics used in eradication campaigns, namely, intradermal tests and interferon-gamma release assay (IGRA), have been deemed of poor specificity because the confirmatory tests (pathological examination and culture) fail to demonstrate the presence of lesions and the involvement of *M*. *bovis* quite frequently [26]. However, disagreements between confirmatory tests and official CMI-based methods are to be expected because their best sensitivity and specificity values are achieved at different immunopathological stages of the infection [26, 27]. Specificities above 99% have been estimated recently for the comparative intradermal skin test using surveillance tests results from officially TB free herds in Great Britain [28]. According to an observational case–control study on confirmed reactors from Northern Ireland, a substantial percentage of non-confirmed reactors could result from imperfect sensitivities of the confirmatory tests [27]. The estimated specificity for the single intradermal test was also high in a study from Spain [29] but infection cannot be confirmed in many slaughtered cattle. This situation raises a lack of confidence of farmers and field veterinarians in official in vivo tests [30]. An epidemiological investigation in Spain pointed out that residual infection and interactions in the wildlife-domestic interface are probably the most relevant causes of bovine TB breakdowns [31]. In order to shed some light on this problem by giving additional reliable explanations for the occurrence of part of the non-confirmed reactor animals, we studied possible differences in the course of experimental TB infection in calves depending on the route of entry of *M*. *bovis* and its implications for diagnosis.

## Materials and methods

### Animal selection

This project was aimed at studying the interference of paratuberculosis vaccination on TB diagnosis [32, 33], the effect of *M*. *bovis* inoculation route on the pathology and diagnostics of bovine TB (the present work) and the efficacy of an inactivated vaccine to protect them from oral and endotracheal challenge with *M*. *bovis* (unpublished work to be submitted). The data presented in this paper were obtained from two control groups belonging to two experimental infections included in the same research project. For this project, thirty, two-month-old animals per experiment were preselected from the same feedlot that had originally purchased them from 13 different farms in northern Spain with no known history of TB. In order to confirm the absence of previous contact with *M. bovis* or other mycobacteria, an IGRA with avian and bovine purified protein derivative standard tuberculins (A-PPD, B-PPD) (CZ Veterinaria, Pontevedra, Spain) was performed in three different samplings at the feedlot. Finally, only 15 animals were recruited and transported to the biosafety level 3 (BSL-3) facilities in NEIKER for each experiment, each with three groups of five calves. In the present work, ten calves were used, five calves from the endotracheal challenge control group (experiment 1) and five calves from the oral challenge control group (experiment 2). Housing conditions, calf keepers and experimental procedures were exactly the same in both experiments.

All the experimental procedures involving animal housing and care were carried out in agreement with the European, National and Regional Law and Ethics Committee regulations. The experimental design underwent ethical review and was approved by NEIKER’s Animal Care and Use Committee (OEBA-NEIKER-2015-010) and by the competent local authority, the Department of Agriculture of Diputación Foral de Bizkaia (PARAPATO-1264-BFA).

### *M. bovis* challenge

After their arrival to the BSL-3 facilities, calves went through a 2-week adaptation period. Afterwards, all calves were challenged with the same *M*. *bovis* field isolate suspended in 2 mL of phosphate-buffered saline (PBS) at the same dose of approximately 10^6^ colony forming units (CFU). The isolate (SB0339 spoligotype profile) was originally obtained from a naturally infected wild boar. This strain has been used in previous experiments at NEIKER and its spoligotype is shared by domestic and wild *M*. *bovis* isolates. The chosen challenge route was different for each of the groups. For the orally challenged group (OC) the infective dose was administered with a syringe and the heads of the animals were maintained upwards until deglutition was observed and no spilling was confirmed. In the group challenged by the endotracheal route (EC), firstly, the needle was introduced in the space between two consecutive tracheal rings located in the range of number 25 and 30 and air was aspirated to assure that the inoculum would be introduced inside the trachea and then the dose was injected. Only EC calves underwent previous intramuscular sedation with XILAGESIC^®^ 2% (10 mg/50 kg) (Laboratorios Calier, S.A., Barcelona, Spain).

### Interferon-gamma release assay (IGRA)

At the BSL-3 facilities, five samplings were performed at 0, 2, 4, 8 and 12 weeks after challenge and an additional one for three EC animals 20 weeks after challenge. Blood was collected from the jugular or caudal vein in tubes with lithium heparin. Stimulation of whole blood with A- and B-PPD as well as with PBS (nil control) was carried out within 8 h of collection. The IDScreen^®^ Ruminant interferon-gamma kit (IDvet, Grabels, France) licensed by the Spanish Government (Ministerio de Agricultura y Pesca, Alimentación y Medio Ambiente) was used for the detection of interferon-gamma in the stimulated blood supernatants according to the manufacturer’s instructions. The standard cut-off of the kit was used to consider a sample positive (i.e., S/P% ≥ 35).

### Skin test

The skin test was carried out in all animals 12 weeks after challenge and it was performed by inoculating 0.1 mL (2500 IU) of A- and B-PPD in the neck. The skin-fold thickness was measured before and 72 h after inoculation. The results of skin thickness increase were interpreted according to the standards of official criteria (EU Council Directive 64/432/CEE and Spanish RD 2611/1996) for both Single Intradermal Test (SIT) and Comparative Intradermal Test (CIT). A calf was considered SIT positive, inconclusive or negative when the increase was 4 mm or greater, between 2 and 4 mm or less than 2 mm, respectively. For CIT, animals were deemed positive, inconclusive or negative when the bovine injection site exceeded the avian site by greater than 4, 1–4 and 1 mm or less, respectively.

### Necropsies

All animals were systematically and thoroughly necropsied. Necropsies from the entire EC group were carried out 12 weeks after challenge. However, animals from the OC group were slaughtered at two different time points. Initially, necropsies began 12 weeks after challenge and gross lesions were absent in the first two animals. Considering that lesions could be at the initial stages of disease and not visible at this time point, the duration of the experiment with the three remaining calves was extended for 8 additional weeks. At the end of the study, animals were sedated by an intramuscular injection of XILAGESIC^®^ 2% (2.5 mg/50 kg) and intravenously injected with T61 (4–6 mL/50 kg) (Intervet International GMBH, Unterschleissheim, Germany). Organs were thoroughly inspected for TB-compatible lesions and collected samples were distributed within five body areas as follows: head (nasal turbinate, palatine tonsils and mandibular, parotid and retropharyngeal lymph nodes (LN), thorax (tracheal, prescapular, tracheobronchial and mediastinal LN), lung (right and left cranial and caudal lobes and medium and accessory lobes), abdomen (hepatic, jejunal and ileocecal LN as well as liver and spleen) and others (prefemoral and popliteal LN).

### Gross pathology

Organs and tissues were visually inspected for the presence of lesions and all of them were thoroughly palpated and sliced in search of deeper lesions. The TB-compatible lesions found were classified according to Palmer et al. [34]. Briefly, two scoring systems were established to measure the severity of the lesions in lung and in LN. The scoring scale for lungs was as follows: 0, no visible lesions; 1, no external gross lesions, but lesions seen upon slicing; 2, less than 5 lesions of < 10 mm in diameter; 3, more than 5 lesions of < 10 mm in diameter; 4, more than 1 distinct gross lesion of > 10 mm in diameter; 5, coalescing gross lesions. Lesion classification of the LN was ranked as follows: 0, no visible lesions; 1, small focal lesion (1–2 mm in diameter); 2, several small foci; 3, extensive lesions.

### Culture

Samples were processed to confirm the presence of mycobacteria in solid (Coletsos-Difco, Francisco Soria Melguizo SA, Madrid, Spain) and liquid culture (BBL Mycobacteria growth indicator tubes (MGIT), Becton–Dickinson, Franklin Lakes, NJ, USA) according to the protocol described previously by Garrido et al. [35]. Briefly, 2 g of tissue was homogenized in 10 mL of sterile distilled water. Five milliliter were decontaminated in hexadecyl-pyridinium chloride 0.75% (w/v) for 12–18 h for solid culture. Samples were centrifuged at 2500 × *g* for 5 min; pellets were cultured in Coletsos tubes at 37 °C for 4 months. The remaining 5 mL were decontaminated and processed for liquid culture in BBL MGIT tubes supplemented with BBL MGIT PANTA and BACTEC MGIT growth supplement according to the manufacturer’s instructions. BBL MGIT tubes were incubated for 42 days in a BACTEC MGIT 960 System.

Colonies were visualized under a stereomicroscope. According to the number of colonies in each tube, a culture score was defined in order to categorize the infection level of each tissue. Score categories were as follows: 0, no growth; 1, less than 10 colonies; 2, between 10 and 50 colonies; 3, over 50 colonies [35].

DNA was extracted from all positive cultures and a *M*. *tuberculosis* complex-specific PCR [36] was performed to confirm that *M. bovis* was responsible for the growth. All isolates were confirmed as *M. bovis* SB0339 by spoligotyping [37].

### Statistics

The number of affected tissues and culture positive samples, as well as scores for gross lesions and cultures were calculated per area and per animal. Differences in the distribution of lesions and positive culture results between groups were assessed using Chi square test. Mann-Whitney U-test was used to study the differences between lesion and culture scores of the different areas of both groups. Correlation between skin test results and lesion scores as well as between skin test results and number of tissues with lesions was performed by Spearman. Statistical significance was considered at *p* values < 0.05. Statistical analysis was completed using R Commander.

## Results

No mortality was recorded during the experiments. All animals completed the trials without showing TB-compatible clinical signs.

### Ante mortem diagnosis: SIT, CIT and IGRA tests

Reactivity to both ante mortem diagnostic techniques was confirmed in both groups (see Table 1). Two weeks after challenge, all EC animals were categorized as reactors to the IGRA test. This positive reactor state remained throughout the experiment for all animals except for one calf that tested negative 12 weeks after challenge. In the case of the OC group, reactivity was first detected 4 weeks after challenge in three of these animals and all five became positive 8 weeks after challenge. However, in the following sampling (12 weeks after challenge) two of them were IGRA negative again.

**Table 1:** **Results for IGRA and skin tuberculin tests performed**

Group	ID	IGRA test						Skin test				
		Weeks after challenge						Δ (mm) skin thickness			Interpretation	
		0	2	4	8	12	20	PPD-A	PPD-B	PPD-B–PPD-A	SIT	CIT
EC	1	N	P	P	P	P	—	1	5	4	P	I
	2	N	P	P	P	P	—	1	12	11	P	P
	3	N	P	P	P	N^a^	—	1	8	7	P	P
	4	N	P	P	P	P	—	0	9	9	P	P
	5	N	P	P	P	P	—	2	9	7	P	P
OC	6	N	N	N	P	P	—	3	16	13	P	P
	7	N	N	N	P	N^b^	—	9	22	13	P	P
	8	N	N	P	P	N^c^	P	3	10	7	P	P
	9	N	N	P	P	P	P	2	8	6	P	P
	10	N	N	P	P	P	P	5	17	12	P	P

SIT: Single Intradermal Test, CIT: Comparative Intradermal Test, EC: endotracheally challenged, OC: orally challenged, Δ: increase, A-PPD: avian PPD, B-PPD: bovine PPD, P: positive, I: inconclusive, N: negative.

^a^ IDscreen S/P% = 28.06.

^b^ IDscreen S/P% = 20.17.

^c^ IDscreen S/P% = 22.26.

—: these animals were necropsied 12 weeks after challenge. The remaining three were tested for IGRA once more before being necropsied 20 weeks after challenge.

On the contrary, all calves from both groups came out as clearly reactors to the SIT and CIT except for one EC calf with an inconclusive CIT result. This animal was exactly at the uppermost skin thickness increase limit (4 mm) to be considered inconclusive but was clearly positive to all IGRA tests from week 2 after challenge on.

Skin test (PPD-B–PPD-A) was negatively although not significantly correlated with lesion scores (rho = −0.446, *p* = 0.197) and the number of tissues with lesions (rho = −0.588, *p* = 0.074).

### Post-mortem analysis

All animals from the EC group presented gross lesions compatible with TB (Table 2). Almost all of the affected tissues found in the EC group (22/28, 79%) were located in the thoracic LN and in the lungs: all five EC animals (5/5, 100%) presented lesions in the thoracic area and three of them (3/5, 60%) also in the lung lobes. Macroscopic lesions in head and abdominal tissues were only found in one of the EC calves (1/5, 20%). Only one affected prefemoral LN was observed and it belonged to an EC animal.

**Table 2:**
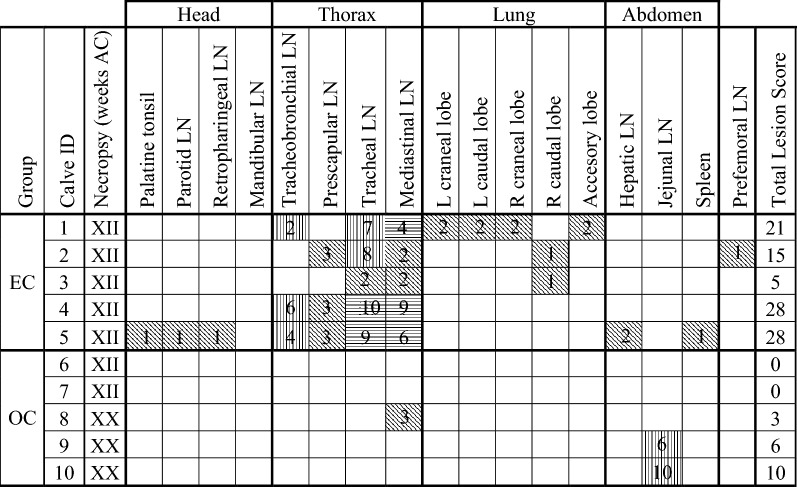
**Distribution and score of confirmed tuberculous lesions in the tissues of the studied groups**

One LN affected (right or left): oblique lines; two LN affected (both right and left or two of three, cranial, caudal, or medial): vertical lines; three LN affected (cranial, caudal, and medial): horizontal lines. Total Lesion score is the sum of the scores of all tissues per animal.

R: right, L: left, EC: endotracheally challenged, OC: oral challenged, AC: after challenge, LN: lymph node.

In the OC group no gross lesions were observed in the two calves slaughtered 12 weeks after challenge. The three remaining OC animals (3/5, 60%) (Table 2) necropsied 20 weeks after challenge presented macroscopic lesions that appeared in two of the five defined areas. In the thoracic region only one affected tissue was detected in one animal. The abdomen was the most affected area (Table 2). More precisely, one of these OC calves showed macroscopic lesions in the proximal, medium or distal jejunal LN while another animal had visible lesions only at the proximal and medium jejunal LN.

Group comparison shows that EC calves presented more macroscopic lesions than OC animals (*p* < 0.001). These differences were also significant for the thoracic and lung areas (*p* < 0.001 and *p* < 0.05, respectively). However, OC animals presented more tissues with macroscopic lesions at the abdomen although differences were not significant and only a tendency was observed (*p* = 0.23).

**Figure 1:**
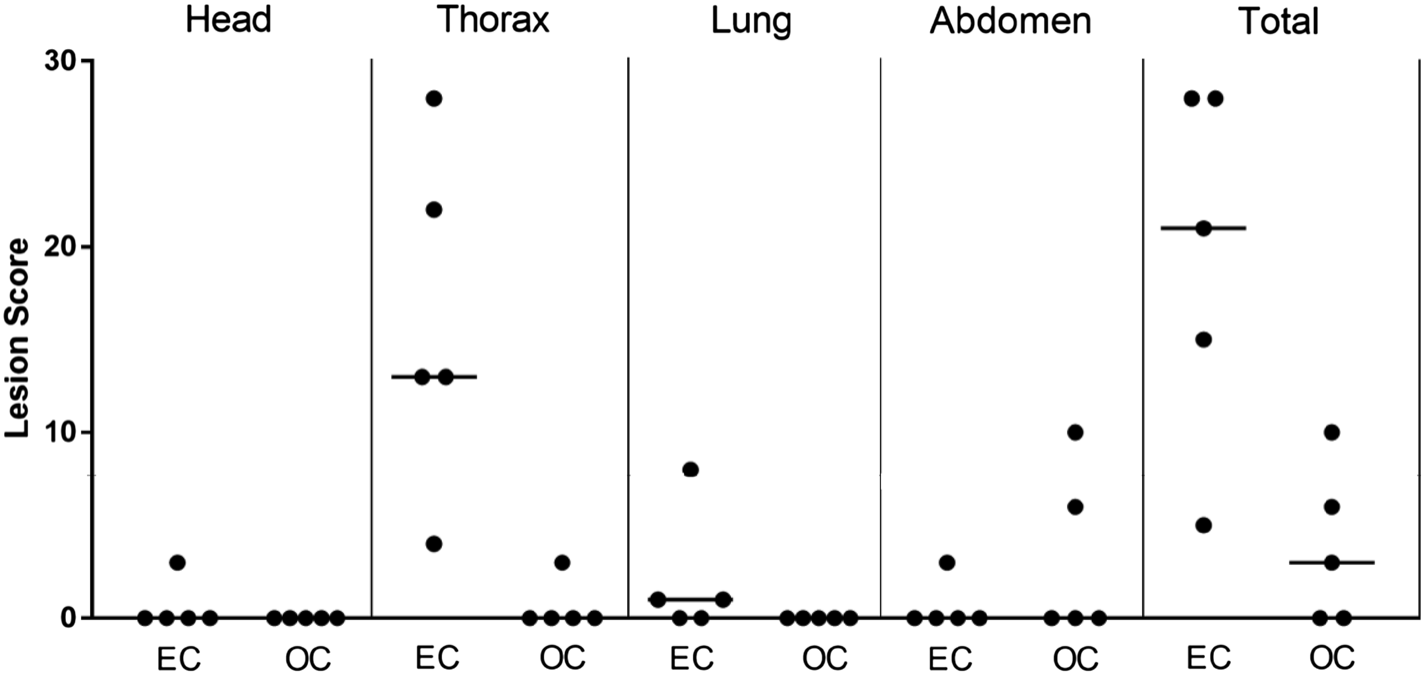
**Tuberculous lesion scores in** ***Mycobacterium bovis*** **challenged calves.** Dot plot representing lesion score distribution in head, thorax, lung, abdomen and total (sum of all areas) for each animal. Horizontal lines show the median values. Significant differences were found in the thorax (*p* < 0.001) and lung (*p* < 0.05). EC: endotracheally challenged, OC: orally challenged.

Lesion scores (shown in Table 2) were always lower for the OC group compared to the EC group in all areas, except for the abdomen (Table 2 and Figure 1), being five times higher in this area for the OC group (16 vs. 3 respectively) (*p* = 0.35). EC animals had significantly higher pathology scores in the thoracic and lung areas (*p* < 0.001 and *p* < 0.05, respectively).

Tissue culture results are shown in Table 3. Culture and/or histopathology confirmed the involvement of *M. bovis* in all tissues presenting gross lesions and additional positive tissues were detected in those showing no macroscopic lesion. In the EC group, more animals (3/5, 60%) yielded positive cultures in the head area than those displaying macroscopic lesions (1/5, 20%). Similarly, in the OC group not only the three animals (3/5, 60%) presenting gross lesions at different sites were confirmed by culture but also the two calves with no macroscopic lesion (5/5; 100%) yielded *M. bovis* isolates from abdominal tissues. EC animals had significantly more positive cultures than OC calves in the thoracic and pulmonary regions (*p* < 0.001 and *p* < 0.05, respectively). Significant differences were also found in relation to the abdominal area where OC animals showed more tissues with positive culture (*p* < 0.05).

**Table 3:**
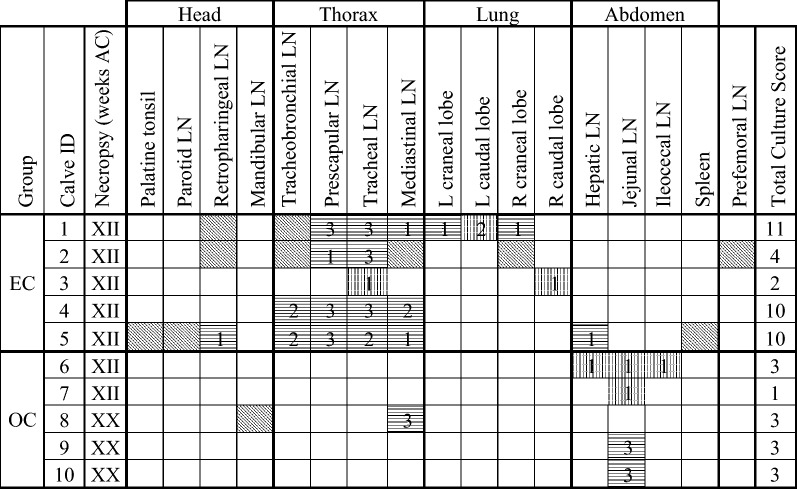
**Distribution of** ***M***. ***bovis*** **positive cultures and culture scores in the tissues of each group**

MGIT culture positive: oblique lines; solid culture positive: vertical lines; both MGIT and solid culture positive: horizontal lines. Culture score is the sum of the scores of all tissues per animal.

R: right, L: left, EC: endotracheally challenged, OC: oral challenged, AC: after challenge, LN: lymph node.

**Figure 2:**
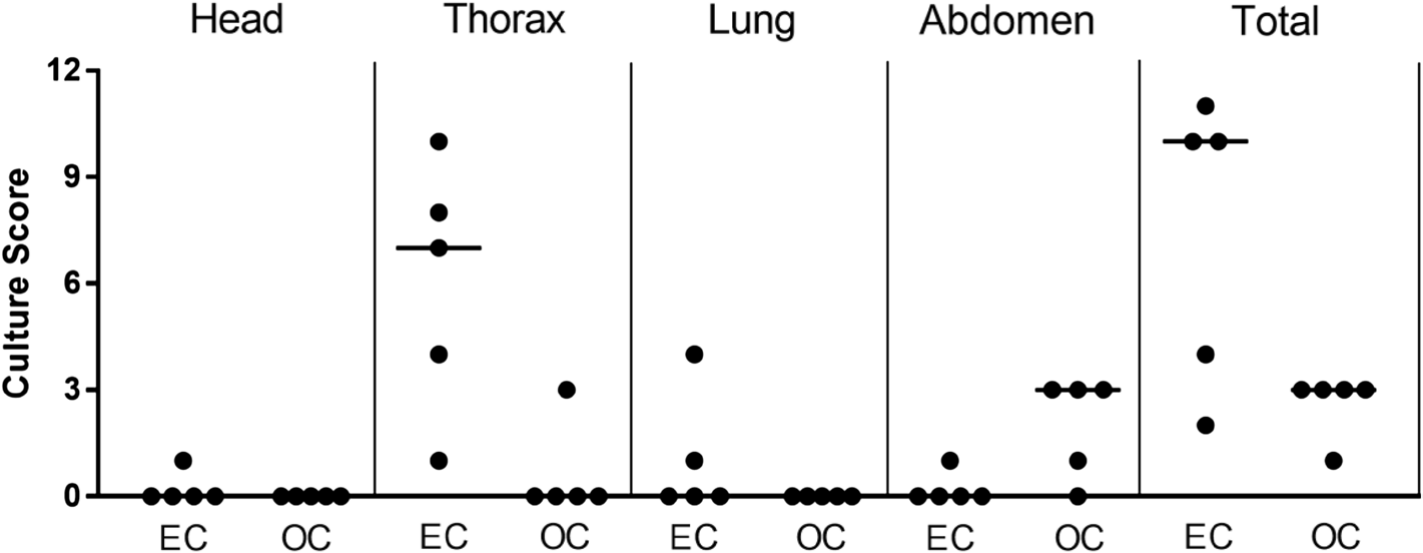
**Culture scores in** ***Mycobacterium bovis*** **challenged calves.** Dot plot representing culture score distribution in head, thorax, lung, abdomen and total (sum of all areas) for each animal. Horizontal lines show the median values. Significant differences were found in the thorax (*p* < 0.05). EC: endotracheally challenged, OC: orally challenged.

Detailed culture scores are shown in Table 3 and Figure 2. Culture scores were always lower in the OC group (thorax *p* < 0.05), except for the abdominal area (Figure 2), where the OC group presented total culture scores ten times higher (10 vs. 1) although significant differences were not observed (*p* = 0.68).

## Discussion

In this experiment, infection was confirmed in all ten animals by culture and histopathology regardless of the challenge route. Despite the small size of study groups, anatomic distribution of gross lesions, culture results as well as pathology and culture scores showed significant differences between both groups. In the EC group, macroscopic lesions and isolation of the etiological agent were observed in all defined areas. However, most of the tissues presenting gross lesions and positive culture, as well as the highest lesion and culture scores, appeared in the thorax and lungs. In contrast, the OC group presented a different picture showing the abdomen as the most affected area, with 4 positive animals out of 5. Furthermore, infection was confirmed by culture in four abdominal LN belonging to the two calves with no visible TB-compatible lesions that were slaughtered at the same time point as the EC group (12 weeks after challenge).

As could be expected [20, 38], these experimental results suggest that depending on the route of infection the distribution of lesions can vary; that is, after an endotracheal challenge, lesions are more likely to be found in the respiratory tract and associated tissues whereas the digestive tract is the most affected area after an oral challenge. Although the number of animals used was small, these findings are consistent with the results obtained in previous studies where it was stated that after inhalation of *M. bovis* most lesions appear in the nasopharynx and lower respiratory tract, including the lungs and associated LN and that *M. bovis* ingestion usually causes lesions in the mesenteric lymph nodes [17, 38, 39]. Moreover, the results obtained by Fitzgerald et al. [21] in tuberculous cows housed together and in calves and cats fed with waste-TB-milk showed the same tendency in the number and distribution of lesions as well as in the areas where positive cultures were detected. Lesions were detected in the thorax in cows infected by airborne transmission and abdominal area for the calves and cats fed with waste milk.

Although the thoracic and lung areas were the primary site of confirmed infection for the EC group, isolation of bacteria from the head, abdominal area or prefemoral LN occurred in three out of five of the animals. This extrapulmonary and extrathoracic dissemination of the etiological agent could be due to oropharyngeal exposure and/or swallowing of tracheobronchial secretions carrying bacteria as mentioned in previous studies [40]. By contrast, the primary affected site in the OC group was the abdomen instead of the thoracic or lung areas. One animal showed no positive culture for the jejunal LN. The same animal was the only one with a positive culture in the thoracic and head areas. The mandibular and mediastinal LN of this animal could have become infected through oropharyngeal exposure and/or inhalation during challenge or with bacteria shed by the other infected OC calves.

According to the outcomes of this experimental study, the time needed for the development of macroscopic lesions seems to differ depending on the infection route. All animals from both groups were clearly reactors to the skin test 12 weeks after challenge, and all five EC calves showed gross lesions during the necropsies. However, lesions were not visible in all OC animals despite positive skin tests and cultures. No macroscopic lesions were found in the two OC calves slaughtered at the same time point as the five EC animals. The three OC calves slaughtered 20 weeks after challenge showed visible lesions. A recent cross-sectional study suggested that tuberculin reaction size (PPD-B–PPD-A size) was significantly positively associated with maximum TB lesion number and size [41]. In contrast to this report studying natural TB cases, we could not see such an association in our experimental setting. In fact, we have seen a negative, although not significant correlation and the greatest tuberculin reaction sizes recorded belonged to the two OC calves only confirmed by *M*. *bovis* isolation from abdominal lymph nodes (Tables 1, 2 and 3). As stated in other studies, the cellular immune response gets activated during the very early stages of the infection [42, 43] and our data suggest that 12 weeks after an oral challenge, the etiological agent may have been able to spread into different abdominal LN as seen by culture results but the time required for visible lesion development seems to take longer.

In line with this, IGRA was able to identify all infected animals in the EC group as early as 2 weeks after challenge. In contrast, the OC group displayed positive animals (3/5) for the first time, 4 weeks after challenge, suggesting that mounting a CMI response capable of producing detectable interferon-gamma levels could need more time in animals infected through the oral route. One EC and two OC animals turned to a negative IGRA status after being positive, but they were clear reactors to the skin test. This phenomenon may simply be related to the performance of the diagnostic kit used or to the dynamics of the immune response to infection of these calves. Despite this, a recent study reported significantly lower sensitivities for IDScreen^®^ compared to the Bovigam^®^ Kit [44]. These authors introduced an additional cut-off point (S/P% ≥ 16) following information provided by the manufacturer [44]. The three samples that turned negative would be deemed positive if this alternative cut-off point was used in our study. Further studies are needed to assess the performance of the different IGRA tests.

We believe that there is not a fully established habit of inspecting tissues from the digestive system as thoroughly as other anatomic sites in abattoirs. Submitting samples from these tissues to the laboratory has not been made common practice unless macroscopic lesions were observed. Taking this into account, in this study we aimed to explore the possibility of attributing a more relevant role to oral transmission in the epidemiology and diagnosis of bovine TB in light of recent research dealing with this widespread wildlife-livestock-multi-host infectious disease [4, 7, 21–25, 31] and the relatively low confirmation rate of skin test reactor cattle [20, 26–28, 30, 41]. Our results demonstrate that the so-called non-confirmed reactors can be composed of animals in early stages of the infection, animals able to contain the spread of bacteria or animals with difficult to detect lesions or lesions in tissues seldom affected and inspected, among others. Reasons for unspecific CMI-based test results have been carefully described in previous reports [26].

These results may contribute to explain the scenario often found in slaughterhouses where animals that are reactors to the ante mortem techniques show no TB-compatible gross lesions during inspection. The sensitivity of visual inspection for lesion detection can be severely compromised because of the difficulty of distinguishing small lesions or infection foci; this issue increases as the dimension of the animal and samples to be checked increases [20]. At population level scales, Byrne et al. [41] showed that a significant proportion of reactor cattle do not present visible lesions at abattoir inspection. In Northern Ireland not all reactor animals are subjected to laboratory testing depending on some epidemiological and pathological parameters [27]. Usually, bulked retropharyngeal, bronchial and mediastinal LN from up to five reactors without macroscopic lesions and at least one lesion from three reactors with visible lesions are submitted to histological examination and/or culture. Under this TB breakdown management strategy, visible lesions were found in 43% of the reactors, although TB could not be confirmed in 0.2% of these cases [27]. On the contrary, the 95.7% of reactors with no visible lesions (the remaining 57% of reactors) could not be confirmed as infected by histology and/or culture of the LN mentioned above. This insensitivity can be accentuated when only the digestive tract is involved in infection. Lesions in this location can readily go unnoticed because these tissues are normally removed at the beginning of slaughtering without being as carefully examined as the thoracic and head tissues. This is of particular relevance in a context of multi-host infection and likely oral inter-species transmission [22, 23].

Although additional research on challenge dose and time required for visible lesion development is necessary, our results indicate that depending on the route of infection, the distribution and development of lesions may vary, and this can have implications for TB diagnostics in terms of confirmation of skin test reactor calves. Therefore, since *M*. *bovis* persists in cattle population and non-confirmed reactors arise, there is an urgent need to improve current control and inspection protocols. Further studies with more animals per group are needed to assess the impact of the route of infection on TB transmission, pathology and diagnosis also under natural conditions.

## Abbreviations

TB: tuberculosis
EC: endotracheally challenged
OC: orally challenged
IGRA: interferon-gamma release assay
LN: lymph node
CMI: cell mediated immunity
SIT: Single Intradermal Test
CIT: Comparative Intradermal Test
PPD: purified protein derivative
A-PPD: avian PPD
B-PPD: bovine PPD
BSL-3: biosafety level 3
PBS: phosphate-buffered saline
CFU: colony forming units
S/P: sample to positive
MGIT: mycobacteria growth indicator tube

## References

[1] Humblet MF, Boschiroli ML, Saegerman C (2009). Classification of worldwide bovine tuberculosis risk factors in cattle: a stratified approach. Vet Res.

[2] Pollock JM, Neill SD (2002). *Mycobacterium bovis* infection and tuberculosis in cattle. Vet J.

[3] Pesciaroli M, Alvarez J, Boniotti MB, Cagiola M, Di Marco V, Marianelli C, Pacciarini M, Pasquali P (2014). Tuberculosis in domestic animal species. Res Vet Sci.

[4] Gortazar C, Che Amat A, O’Brien DJ (2015). Open questions and recent advances in the control of a multi-host infectious disease: animal tuberculosis. Mamm Rev.

[5] Olea-Popelka F, Muwonge A, Perera A, Dean AS, Mumford E, Erlacher-Vindel E, Forcella S, Silk BJ, Ditiu L, El Idrissi A, Raviglione M, Cosivi O, LoBue P, Fujiwara PI (2016). Zoonotic tuberculosis in human beings caused by *Mycobacterium bovis*-a call for action. Lancet Infect Dis.

[6] Caminiti A, Pelone F, LaTorre G, De Giusti M, Saulle R, Mannocci A, Sala M, Della Marta U, Scaramozzino P (2016). Control and eradication of tuberculosis in cattle: a systematic review of economic evidence. Vet Rec.

[7] Good M, Bakker D, Duignan A, Collins DM (2018). The history of in vivo tuberculin testing in bovines: tuberculosis, a “One Health” issue. Front Vet Sci.

[8] de Kantor IN, Ritacco V (2006). An update on bovine tuberculosis programmes in Latin American and Caribbean countries. Vet Microbiol.

[9] Essey MA, Koller MA (1994). Status of bovine tuberculosis in North America. Vet Microbiol.

[10] Radunz B (2006). Surveillance and risk management during the latter stages of eradication: experiences from Australia. Vet Microbiol.

[11] Rivière J, Carabin K, Le SY, Hendrikx P, Dufour B (2014). Bovine tuberculosis surveillance in cattle and free-ranging wildlife in EU Member States in 2013: a survey-based review. Vet Microbiol.

[12] Collins JD (2006). Tuberculosis in cattle: strategic planning for the future. Vet Microbiol.

[13] Gordejo FJR, Vermeersch JP (2006). Towards eradication of bovine tuberculosis in the European Union. Vet Microbiol.

[14] Napp S, Allepuz A, Mercader I, Nofrarias M, Lopez-Soria S, Domingo M, Romero B, Bezos J, Perez de Val B (2013). Evidence of goats acting as domestic reservoirs of bovine tuberculosis. Vet Rec.

[15] Muñoz-Mendoza M, Romero B, Del Cerro A, Gortazar C, Garcia-Marin JF, Menendez S, Mourelo J, de Juan L, Saez JL, Delahay RJ, Balseiro A (2016). Sheep as a potential source of bovine TB: epidemiology, pathology and evaluation of diagnostic techniques. Transbound Emerg Dis.

[16] Di Marco V, Mazzone P, Capucchio MT, Boniotti MB, Aronica V, Russo M, Fiasconaro M, Cifani N, Corneli S, Biasibetti E, Biagetti M, Pacciarini ML, Cagiola M, Pasquali P, Marianelli C (2012). Epidemiological significance of the domestic black pig (*Sus scrofa*) in maintenance of bovine tuberculosis in Sicily. J Clin Microbiol.

[17] Domingo M, Vidal E, Marco A (2014). Pathology of bovine tuberculosis. Res Vet Sci.

[18] Liebana E, Johnson L, Gough J, Durr P, Jahans K, Clifton-Hadley R, Spencer Y, Hewinson RG, Downs SH (2008). Pathology of naturally occurring bovine tuberculosis in England and Wales. Vet J.

[19] Neill SD, Skuce RA, Pollock JM (2005). Tuberculosis—new light from an old window. J Appl Microbiol.

[20] Gormley E, Corner LAL (2017). Pathogenesis of *Mycobacterium bovis* infection: the badger model as a paradigm for understanding tuberculosis in animals. Front Vet Sci.

[21] Fitzgerald SD, Hollinger C, Mullaney TP, Bruning-Fann CS, Tilden J, Smith R, Averill J, Kaneene JB (2016). Herd outbreak of bovine tuberculosis illustrates that route of infection correlates with anatomic distribution of lesions in cattle and cats. J Vet Diagn Invest.

[22] Barasona JA, Vicente J, Diez-Delgado I, Aznar J, Gortazar C, Torres MJ (2017). Environmental presence of *Mycobacterium tuberculosis* complex in aggregation points at the wildlife/livestock interface. Transbound Emerg Dis.

[23] Cowie CE, Hutchings MR, Barasona JA, Gortazar C, Vicente J, White PCL (2016). Interactions between four species in a complex wildlife: livestock disease community: implications for *Mycobacterium bovis* maintenance and transmission. Eur J Wildl Res.

[24] Santos N, Santos C, Valente T, Gortazar C, Almeida V, Correia-Neves M (2015). Widespread environmental contamination with *Mycobacterium tuberculosis* complex revealed by a molecular detection protocol. PLoS One.

[25] Santos N, Almeida V, Gortazar C, Correia-Neves M (2015). Patterns of *Mycobacterium tuberculosis*-complex excretion and characterization of super-shedders in naturally-infected wild boar and red deer. Vet Res.

[26] de la Rua-Domenech R, Goodchild AT, Vordermeier HM, Hewinson RG, Christiansen KH, Clifton-Hadley RS (2006). Ante mortem diagnosis of tuberculosis in cattle: a review of the tuberculin tests, gamma-interferon assay and other ancillary diagnostic techniques. Res Vet Sci.

[27] O’Hagan MJ, Courcier EA, Drewe JA, Gordon AW, McNair J, Abernethy DA (2015). Risk factors for visible lesions or positive laboratory tests in bovine tuberculosis reactor cattle in Northern Ireland. Prev Vet Med.

[28] Goodchild AV, Downs SH, Upton P, Wood JL, Rua-Domenech R (2015). Specificity of the comparative skin test for bovine tuberculosis in Great Britain. Vet Rec.

[29] Alvarez J, Perez A, Bezos J, Marques S, Grau A, Saez JL, Minguez O, de Juan L, Dominguez L (2012). Evaluation of the sensitivity and specificity of bovine tuberculosis diagnostic tests in naturally infected cattle herds using a Bayesian approach. Vet Microbiol.

[30] Ciaravino G, Ibarra P, Casal E, Lopez S, Espluga J, Casal J, Napp S, Allepuz A (2017). Farmer and veterinarian attitudes towards the bovine tuberculosis eradication programme in Spain: what is going on in the field?. Front Vet Sci.

[31] Guta S, Casal J, Napp S, Saez JL, Garcia-Saenz A, Perez de Val B, Romero B, Alvarez J, Allepuz A (2014). Epidemiological investigation of bovine tuberculosis herd breakdowns in Spain 2009/2011. PLoS One.

[32] Serrano M, Elguezabal N, Sevilla IA, Geijo MV, Molina E, Arrazuria R, Urkitza A, Jones GJ, Vordermeier M, Garrido JM, Juste RA (2017). Tuberculosis detection in paratuberculosis vaccinated calves: new alternatives against interference. PLoS One.

[33] Serrano M, Elguezabal N, Sevilla IA, Geijo MV, Molina E, Juste RA, Garrido JM (2017). Preliminary results indicate that inactivated vaccine against paratuberculosis could modify the course of experimental *Mycobacterium bovis* infection in calves. Front Vet Sci.

[34] Palmer MV, Thacker TC, Waters WR (2007). Vaccination of white-tailed deer (*Odocoileus virginianus*) with *Mycobacterium bovis* bacillus Calmette Guerin. Vaccine.

[35] Garrido JM, Sevilla IA, Beltran-Beck B, Minguijon E, Ballesteros C, Galindo RC, Boadella M, Lyashchenko KP, Romero B, Geijo MV, Ruiz-Fons F, Aranaz A, Juste RA, Vicente J, de la Fuente J, Gortazar C (2011). Protection against tuberculosis in Eurasian wild boar vaccinated with heat-inactivated *Mycobacterium bovis*. PLoS One.

[36] Sevilla IA, Molina E, Elguezabal N, Perez V, Garrido JM, Juste RA (2015). Detection of mycobacteria, *Mycobacterium avium* subspecies, and *Mycobacterium tuberculosis* complex by a novel tetraplex real-time PCR assay. J Clin Microbiol.

[37] Kamerbeek J, Schouls L, Kolk A, van Agterveld M, van Soolingen D, Kuijper S, Bunschoten A, Molhuizen H, Shaw R, Goyal M, van Embden J (1997). Simultaneous detection and strain differentiation of *Mycobacterium tuberculosis* for diagnosis and epidemiology. J Clin Microbiol.

[38] Pollock JM, Rodgers JD, Welsh MD, McNair J (2006). Pathogenesis of bovine tuberculosis: the role of experimental models of infection. Vet Microbiol.

[39] Collins CH, Grange JM (1983). The bovine tubercle bacillus. J Appl Bacteriol.

[40] Perez de Val B, Lopez-Soria S, Nofrarias M, Martin M, Vordermeier HM, Villarreal-Ramos B, Romera N, Escobar M, Solanes D, Cardona PJ, Domingo M (2011). Experimental model of tuberculosis in the domestic goat after endobronchial infection with *Mycobacterium caprae*. Clin Vaccine Immunol.

[41] Byrne AW, Graham J, Brown C, Donaghy A, Guelbenzu-Gonzalo M, McNair J, Skuce RA, Allen A, McDowell SW (2018). Modelling the variation in skin-test tuberculin reactions, post-mortem lesion counts and case pathology in tuberculosis-exposed cattle: effects of animal characteristics, histories and co-infection. Transbound Emerg Dis.

[42] Ritacco V, Lopez B, de Kantor IN, Barrera L, Errico F, Nader A (1991). Reciprocal cellular and humoral immune responses in bovine tuberculosis. Res Vet Sci.

[43] Welsh MD, Cunningham RT, Corbett DM, Girvin RM, McNair J, Skuce RA, Bryson DG, Pollock JM (2005). Influence of pathological progression on the balance between cellular and humoral immune responses in bovine tuberculosis. Immunology.

[44] Casal C, Infantes JA, Risalde MA, Diez-Guerrier A, Dominguez M, Moreno I, Romero B, de Juan L, Saez JL, Juste R, Gortazar C, Dominguez L, Bezos J (2017). Antibody detection tests improve the sensitivity of tuberculosis diagnosis in cattle. Res Vet Sci.

